# The logic of medical reasoning: toward an integrated inductive, deductive, and abductive approach to clinical practices

**DOI:** 10.1186/s13010-025-00178-y

**Published:** 2025-09-17

**Authors:** Ahti-Veikko Pietarinen, Donald E. Stanley

**Affiliations:** 1 Hong Kong Baptist University, Hong Kong, China; 2 Maine Medical Center, Portland, Maine, United States

**Keywords:** Logic and reasoning, Philosophy of diagnostics, Abduction in medical reasoning, Deduction, Induction, Noise hygiene

## Abstract

**Introduction:**

This study explored the logical underpinnings of medical reasoning, focusing on the integration of abduction, deduction, and induction within clinical decision-making. It aimed to highlight the role of abduction in generating hypotheses, particularly in complex cases that defy standard protocols, and to examine the synergy between human expertise and AI-assisted tools in enhancing diagnostic accuracy.

**Methods:**

The research employed a qualitative approach, analyzing philosophical theories and integrating them with clinical case studies. The study examined the interplay of logical processes in medical diagnostics and the application of abduction in rare and novel cases. Additionally, the potential of AI-assisted tools to support clinical reasoning and reduce diagnostic noise was explored.

**Results:**

Abduction was identified as a critical yet often underappreciated element in medical reasoning essential for hypothesis generation. Deduction refines hypotheses against established medical knowledge, while induction validates decisions through empirical data. AI-assisted tools were found to enhance diagnostic accuracy by reducing noise, although they did not engage in the musement or genuine abductions that characterize human clinical reasoning.

**Discussion:**

The study concluded that a triadic approach to clinical reasoning, incorporating abduction, deduction, and induction, is essential for effective medical diagnostics. In particular, abduction plays a pivotal role in navigating the complexities of clinical decision-making. The integration of AI tools can reduce noise and improve diagnostic processes, but the essential human elements of insight and judgment remain irreplaceable in patient care.

## Introduction

Medicine is a discipline characterized by a rich interplay of logical processes, each of which is critical at various stages of patient care [16, 37, 42]. Although Bayesian reasoning is a well-known method in diagnostic processes and in interpreting test outcomes, frequentist statistics underpin the design and analysis of randomized controlled trials (RCTs; [1, 23, 70]), informing therapeutic decisions and outcome interpretations.

Despite all their advances, both Bayesian and frequentist methods are limited to only one out of three types of reasoning, namely, inductive inference. However, the entire setting of medical reasoning in individual, personal, and institutional contexts is a much wider realm [69, 71]. Induction directs choices and sequences of tests, but the process of making diagnoses begins not with induction and statistical reasoning methods but with forming the bedrock of the diagnostic process – this is abduction – from which the process could be sensibly and reasonably oriented toward successful outcomes in the hope of preventing unnecessary and unexpected diagnostic odysseys. The process of abduction involves determining how and which working hypotheses are to be formulated first.

Together with abduction, we need to consider both induction and deduction. Sandwiched between abduction and induction is deduction, which is employed to demonstrate the predictive value of abductive recommendations, such as validating the efficacy of treatment guidelines and protocols. Induction is the third and last stage of clinical reasoning, according to the venerable procedure that follows the order of reasoning in scientific discovery. These orders, including the term ‘abduction’, come from the writings of American logician and polymath Charles S. Peirce [41].

In this paper, we integrate medical reasoning with the art of informed guessing, known as abduction, to navigate the intricacies of clinical decision-making. First, we examine the interplay of these logical processes in the overall context of medical diagnostics. We discuss risk perception in the fallible context of hypothesis generation and test selection throughout the diagnostic process, and address the application of abduction in the face of rare and novel cases. Three case scenarios illustrate the practical application of these reasoning methods in clinical and diagnostic settings. We approach the acquisition of medical knowledge in the middle of things: between *contexts*, clinical *presentations*, and patients’ *experiences*, without definite beginning and end points. We highlight the importance of triadic relationships and operations of thought in physicians, patients, and institutions, which shape diagnostic and therapeutic strategies in decision making in institutionalized settings (cf. the triadic model of clinical epistemology in [28]). Our intention is to provide an integrated picture of the logical underpinnings of clinical reasoning and its implications for medical practice.

## Abduction, deduction, and induction in medical reasoning

Abduction, or informed guessing in professional and scientific contexts, is a term coined by Peirce in the late nineteenth century [4, 10, 22, 41, 43–45]. It is a critical yet often underappreciated element of medical reasoning. Although the role of abduction in clinical decision-making has been acknowledged rather well [3, 8, 9, 13, 14, 21, 31, 34, 46, 56–59, 62, 64, 65, 67, 68], its place and integration in the medical decision-making process in the overall institutional context have been less studied.

Abduction involves formulating preliminary questions based on clinical presentations, impressions, and interviews, aiming at corroborating questions with patient history, physical examination, and supporting data. This diagnostic process culminates in actionable recommendations, which are hypotheses. Abduction thus also encompasses not only the generation of novel hypotheses in generic diagnostic contexts. It is also the type or inference that is of particular utility when professionals are faced with clinical problems that cannot be resolved well by the existing guidelines, checklists, and clinical pathways, as is often the case when diagnosing rare, extremely rare, hyperrare, or even altogether unique and novel cases.

Deduction is interwoven with the processes of induction and abduction to form a robust framework for clinical decision-making. It is characterized as the logical mechanism through which physicians validate and refine their hypotheses, draw upon established medical knowledge, interpret the weight of the totality of evidence (such as clinical testing results), and apply clinical guidelines to infer the most likely diagnoses or treatment pathways. This step follows the generation of an initial hypothesis through abduction. Deduction in medical reasoning serves as a critical checkpoint, ensuring that the conclusions drawn from patient data and potential diagnoses are consistent with known medical science. It is not only a passive analytical tool devoid of content but also an active participant in the iterative process of medical problem solving, as it helps to confirm or refute the physician’s preliminary insights with rigor and clarity.

Induction is the third stage of clinical reasoning, where inferences are drawn from test results and examination findings. A variety of methods and theories, including Bayesian and frequentist statistical methods, may be available. The interpretation of the results of the inductive procedure involves an internal dialogue, where the physician or the team weighs on the implications of positive or negative test outcomes. This process involves making judgments and is typically depicted by a 2 × 2 table of test strategies consisting of true positives (patients who tested positive for disease), true negatives (patients without disease testing negative), false positives (patients who tested positive without disease), and false negatives (patients with disease testing negative).

All three elements of medical reasoning are important and should not be treated and addressed in isolation from one another. Dealing with low and extremely low prestudy odds in discovery-led research is analogous to dealing with presentations of rare diseases, where the exploitation of abductive reasoning abilities shines above all others. In more mechanical cases of handling complicated differentials, induction and statistical procedures are of pronounced importance, as they involve the verification and testing of particular ideas, hypotheses and treatments. By employing different stages of reasoning as an integrated instrument, clinicians can systematically eliminate possibilities, streamline diagnostic processes, and apply therapeutic principles with both precision and novelty, ultimately guiding patient care toward evidence-based outcomes even when presented with rare and unusual cases.

## Risk perception *in media res:* clinical diagnostic uncertainties

Clinical diagnostics is an empirical science that begins with the observation of significant and surprising events, leading to hypothesis generation and validation of informed guesses through further reasoning cycles. It is rooted in the same uncertainties and epistemic ignorance as scientific methods are [19, 36], yet it is not infallible. Judgments about diagnoses, treatments, and prognoses evolve as scientific understanding advances, medical education improves, and disease manifestations are increasingly uncovered.

From the point of view of clinical practice, selecting pertinent stimuli from patients’ complaints, signs, symptoms, and physical examinations is not a rigorously defined process; it requires a balance between premature closure and excessive data gathering. Experience guides clinicians in retaining or discarding observations, allowing for contemplation of the findings.

Clinical and medical reasoning begins not *ab ovo* but with the *res media*—the middle of things. The first patient encounter is book-ended by observation. Clinicians convert sensory inputs, such as sweating, fever, and pain, into potential diagnoses via self-reflection and self-examination of mental irritations via stimuli that cause beliefs, hunches, and potential meanings to arise: diaphoresis may be observed in hot climates, shock, drug reactions, elevated cortisol levels, insomnia, and embarrassment. All this happens in the context of reasoning, cultures, routines, habits and careers developing over decades, in interplay with new and emerging technologies.

For instance, ductal carcinoma in situ may be detectable by mammography, but not all such tumors progress to invasive cancer if a protective factor surrounds the breast stroma and a potential inherent in the epithelial cells does not transgress to the basement membrane to invade the stroma. Moreover, other types of breast cancer may be undetected by this method. Similarly, certain lung cancers may develop resistance to conventional treatments, necessitating a re-evaluation of the ‘positive predictive value’ of diagnostic methods and a shift toward personalized medicine based on genomic variability.

The natural back-and-forth between doubt and belief in reasoners’ minds is informed by past experiences and training, which provide the categories for interpretation. Immanuel Kant recognized how filtered classification of cognition and reasoning begins in the workings of one’s sensory and cerebral mechanisms; Peirce wrote on “our senses as reasoning machines” – modern cognitive science will wholeheartedly agree on such fundamental points.

The engagement of medicine with readily interpreted sensory and machine-curated algorithmic data equally necessitates an acknowledgment of fallibilism — the possibility of error and heuristic deficiencies [30, 72]. For example, the evolution of cancer treatment from nonspecific cytotoxic drugs to targeted therapies such as monoclonal antibodies and hormone manipulation reflects a significant shift in oncological strategies, influenced by the emergence of genomics. In addition, there are several environmental and contextual factors that influence how treatment may proceed.

Observation is the cornerstone of diagnosis. With our seven senses – including proprioception as well as our criticized, justified, and validated intuition-based mechanisms for judgment – imaginative inquiry is facilitated. It begins with abduction and with its “play of musement” (free play of ideas, or how to notice a novelty or anomaly), aptly described by Peirce as that reflective contemplative state of mind that precedes successful abductions and idea generation [15, 51]. While using AI-assisted diagnostics and its recognized role in reducing noise and hence errors, machines engage neither in the musement nor in the genuine abductions [29, 35, 48]. We will illustrate the importance of abduction in terms of three case scenarios and then return to the topic of the use of AI in medical diagnostics.

## Discussion and three case scenarios

### Key elements in the diagnostic steps

The diagnostic step is inherently creative, importantly involving abduction, which introduces promising courses of differential diagnosis, leading to hypothesis testing to determine proper therapeutic options. The stratification of diagnoses based on symptom acuity is crucial. Symptoms and observations generate hypotheses, which are then ordered based on likelihood and assessed accordingly. The practice of diagnosis, or the mastery of reasoning, often involves making educated guesses based on experience, which informs the prior probabilities used in Bayesian reasoning in the later stages of the procedure.

For example, it follows from observations that if there is chest pain radiating to the shoulders and that if this symptom alone is present, it may signal myocardial infarction, acute pancreatitis, or pulmonary embolism. However, if an infarct is highly suspected, then the hypothesis should be tested with enzyme measurement and ECG tracing. Thus, the practice of diagnosis must also be stratified based on acuity: symptoms and the portending consequences, e.g., acute myocardial infarction.

Symptoms are elicited, and signs are observed to form a clinical picture: the presence of arrhythmia, pulsus paradoxus, a decrease in blood pressure, and early signs of shock, for instance, can be indicative of underlying pathologies. These clinical manifestations prompt the generation of diagnostic hypotheses. The subsequent challenge lies in prioritizing these hypotheses based on their explanatory power regarding the observed signs and symptoms. This prioritization is guided by the acuity of the presentation and the estimated likelihood of each condition, known as the prior probability, which then informs the selection of appropriate diagnostic tests.

The deductive reasoning process, as famously exemplified by Sherlock Holmes (as, say, the case of nonbarking dog) but in truth concerning one’s abductive faculties of making informed guesses and selections among those [31], involves deriving strong suspicions about the veracity of a hypothesis from keen observation – or should do so even when the causal link between the observation and the hypothesis is not immediately apparent or not recoverable at all. Correct guesses are often informed by experience, which refines our ability to estimate posterior probabilities; outcomes that, once observed, inform future prior probabilities in medical practice. This iterative process is a cornerstone of Bayesian reasoning.

Peirce, who coined the term abduction to refer to the creative initiation of conjecture-making in scientific inquiry, emphasized the principle of economy in the trustworthy and fruitful workings of abductive reasoning [11]. In abduction, economy means to advocate for the judicious use of resources – money, time, thought, and energy – to guide the investigative process. The principle of economy encourages a move toward experimental action. Relying on these principles enhances reasoning, costly and time-consuming both in experimental scientific inquiry and in clinical practice. The challenge in clinical diagnosis is to judiciously narrow down the list of potential diagnoses by assessing the likelihood of each, informed by clinical experience, the patient’s context, and their medical history. The choices are also constrained by considerations of economy, prompting further urgency to the reasoning abilities of professionals to set the initial diagnoses on the right track.

It is crucial to avoid prematurely discounting potential diagnoses, as this limits the scope of “musement”, which involves dissecting observations into manageable segments that can be economically tested within the clinical setting. For example, through a further review of signs and symptoms or the use of readily available diagnostic tools such as handheld ultrasounds, we can manage the orientation of the course of diagnostic reasoning with intermittent input data. Each hypothesis can then be scrutinized through deductive reasoning, asking “what if…?” and proceeding to test only those hypotheses that withstand critical evaluation. This process can helpfully involve machine reasoning. After that, the reasoning cyclically returns to the initial examination, refining perceptions and associated abductive inferences through controlled iterations and interactions between the physician, patient, and institutional routines.

### Three case scenarios

We present three case scenarios that illustrate these elements of clinical reasoning in their specific ways.

### Case scenario A:

1. A 54-year-old man is brought to the emergency room of a hospital and is seen by a triage nurse with the complaint of sudden-onset severe chest pain. The nurse inserts a catheter to administer fluids and medications.
2. Patient: I was descending stairs on my way to breakfast when I experienced severe chest pain (scale 7/10)
3. Nurse: How often have you had this type of chest pain?
4. Patient: I have never had this pain before. I need help!
5. Physicians recommend the administration of 10 mg of morphine through intravenous lines. The patient was considered to have acute coronary syndrome, including angina pectoris and myocardial infarction; these conditions were ruled out as emergency cases.
6. Patient: I feel less pain now. (His blood pressure decreased to 100/65 mmHg.)
7. Blood pressure monitoring and oxygen administration are standard approaches for evaluating chest pain.
8. Physician orders fluids, cardiac enzymes, and ECG stats. They quickly recognize that the condition is urgent. During physical examination, the doctor hears a soft, holosystolic cardiac murmur grade 2/6 over the precordium.
9. Emergent vascular imaging (MRI) revealed enlargement of the descending aorta and expansion of the aortic arch.
10. Medical anti-impulse therapy for type B dissecting aortic aneurysms involves the use of beta-blockers to reduce the heart rate to less than 60 bpm.
11. The patient’s family history was positive for vascular Ehlers‒Danlos syndrome, and family screening was initiated.
12. A plan for the use of a thoracic stent graft was established. The patient’s condition stabilized.

### Case scenario B:

A 57-year-old male presented with cognitive decline and, for the first time, multiple tonic‒clonic seizures. EEG, CSF serology, angiogram, and whole-body PET scans were negative until MRI in 2015 revealed a single dot-like lesion in the right hippocampal head. Neurocognitive decline continued until expected death approximately 14 years after initial symptom onset.

To perform differential diagnosis, one needs to consider records of drug usage and extensive family history, as well as the elimination of all known causes, including idiopathic causes, epilepsy, all primary vascular diseases such as angiitis, berry aneurysm, AV malformation, microembolism, remote microinfarct, dermoid/epidermoid cyst, Sturge‒Weber syndrome, chronic encephalitis, early-onset dementia, tuberous sclerosis, ganglioglioma, and metabolic diseases such as gangliosidoses such as Batten’s disease, Fabry’s disease, other CLNN types 1, 2, and 3, enzyme analysis of palmitoyl-protein thioestereae (PPT)−1 and tripeptidyl-peptidase (TTP)−1 genetic recessive abnormalities, and a myriad of other storage diseases.

With this patient, brain autopsy revealed luxol-PAS-positive deposits within neurons, confirming Kufs disease of the *adult neuronal ceroid lipofusinosis* class. Similar deposits may be identified in the rectal mucosa [2, 5].

### Case scenario C:

A 67-year-old woman presented in the clinic who seemed behaviorally ‘changed over the summer’ from congenial and engaged to intolerant, avoiding contact, and stumbling. With respect to abnormal gait, one would typically consider the early onset of Alzheimer’s or Parkinson’s disease. MRI revealed a normal brain volume and no suspicion of multi-infarct dementia but a very subtle indistinct cortical white matter pattern.

At autopsy, there were few plaques, few amyloid and tau deposits, and occasional fibrillary tangles. None of the patients with this cause were confidently diagnosed with dementia or other neurogenerative conditions. The left cortex, however, contained several areas of vacuolar degeneration in the white matter. A few areas of gliosis and Western blot characteristics were suggestive of disease-specific PrP^Sc^ proteins, which are elements of the infectious prion agent Scrapie (final diagnosis: Creutzfeldt‒Jakob Disease (CJD); [47].

### Generic lessons from the case scenarios

In the case of chest pain (Case A), the diagnostic process begins with a dialogue between the patient and the physician. A list of differential diagnoses is constructed, informed by frequentist disease probability and Bayesian considerations of prior probabilities and updates on conditional probabilities of the hypothesis given the evidence. Each test in the emergency clinic is ordered to aid in hypothesis generation and the inductive process of diagnosis refinement through rigorous testing. The test or family history, when available for the genetic basis of vascular Ehlers–Danlos syndrome, is highly sensitive and specific for this condition [55].

Experience significantly enhances the diagnostic process. For instance, a physician practicing in a foreign country may encounter diseases uncommon in their home country, such as Chagas disease, which is rare in the United States but more prevalent in Latin America. Bayesian logic can assist in testing for such diseases in patients with relevant travel histories. These sensory irritations also serve as invitations to doubt, requiring the clinician to question initial conjectures by consulting one’s senses in a self-reflective and self-aware manner.

Case B concerns neuropathology, in which the accumulation of amino acids and lipids occurs not only in cerebral white matter but also, surprisingly, in the rectal mucosa [2, 5]. Biopsy of the rectal mucosa or NGS genomics can be ordered and added to a rapid diagnosis when sufficient suspicion has arisen [53].

Regarding Case C, in these extremely rare prion diseases, white matter patterns may remain subtle and indistinct compared to those in noninfectious neurogenerative cases.

### Methodological observations

In everyday practice, clinicians seek “an explanation of a particular fact by finding some salient features of the particular that allow it to be explained by some more general causal principles” [4, 44, 50].

Abductive reasoning is a natural working of the rational mind to look for sensible explanations. Representations of key data points and correlations arising from statistical and linguistic data are not only linguistic but also exploit visual, diagrammatic, and other nonlinguistic clues and modalities that may help guide imagination and improve the skill and accuracy of reasoning in integrated professional-client (doctor‒patient) encounters. Drawing new diagrams in new domains of medical and other data creates opportunities to better automate hypothesis generation when proper qualities for diagrammatic representation are selected (for example, PAD lesions represent asymmetries, border smoothness and other morphological features).

There are at least three reasons why improving diagnostic skills concerning rare and extremely rare cases is important. First, primary care practitioners tend to overestimate the prevalence of their preferred diagnoses in both the pre- and posttest stages [38], increasing the probability of prematurely ruling out rarer cases in the long tails of the probability distribution. Second, the prevalence of extremely rare cases could in fact be high: Smith et al. [54] estimated that more hyperrare diseases (affecting < 1/10^8^ individuals) prevail per capita than rare diseases, constituting 1/10 of all human illnesses altogether [20]. Third, the average diagnostic age for rare diseases from first symptom onset to accurate diagnosis varies from 5 to 19 years for adult patients and up to 6 years for children [6, 27]. New methods to expedite such processes at all levels of healthcare systems, including re and upskilling, are clearly valuable. The development of these methods began with identifying proper theoretical frameworks for AI-assisted medical reasoning and practice.

## Reducing noise by AI in medical reasoning

We asked two questions. What do AI-assisted diagnostic tools and programs add to inquiry-led medical reasoning? Second, can machines perform abduction, in addition to their commonplace computation (deduction) and machine learning (induction)?

First, if we approach our diagnostic decision-making processes at the systemic and institutional levels to resolve problematic situations, AI tools can be of significant help. They can considerably improve accuracy by reducing diverse types of noise in human decision making by following a recognized decision hygiene procedure [25, 52]. Boyle et al. [7] noted the phenomenon of context specificity in diagnostics, which “refers to the vexing phenomenon whereby a physician can see two patients with the same presenting complaint, identical history and physical examination findings, but due to specific situational (contextual) factors arrives at two different diagnostic labels.” This is what Kahneman et al. [25] referred to as *occasion noise*, and it can occur with the teams of clinical reasoners as well as individual physicians.

Significant interpersonal noise and low kappa values persist in pneumonia diagnoses by radiologists and pathologists: “variation in skill can explain 44% of the variation in diagnostic decisions” regarding pneumonia [: 247]. Likewise, there was significant variability in evaluating angiographs on whether major vessels were 70% blocked, the number and location of endometriotic lesions, and the diagnostic accuracy of melanoma from skin biopsies (with only 64% accuracy). The prevalence of false negatives in breast cancer screenings from mammographs varies from 0 to 50% from physician to physician [25,25: 250]. Such variance can take the form of occasion noise, meaning that the same physician or diagnostician will exhibit such variability for the exact same data when presented at different times of the day or on different dates.

Reducing all manifestations of noise through diagnostic and decision-making guidelines will have a significant impact on reducing diagnostic errors caused by factors related to human cognition (cf. [25, 28, 33, 63, 73]). Proper decision hygiene to reduce noise prevents unexpected errors and adverse incidents before their onset.

The AI-supported diagnostic reasoning framework is also helpful in reducing *group-level noise*: in human groups (e.g., hospital teams of doctors), some individuals dominate the discussion (potentially suffering from the Dunning-Kruger effect); they might be given unwanted or nonblinded cues that influence other members’ judgment skills in an adversarial fashion (simply the dressing and manners matter), the order of information presented matters (especially when panels vote), groups become polarized and vulnerable to information cascades, and they are less receptive to experimental and unconventional proposals from other (junior) colleagues, having group-level blind spots, and so on. None of these factors should play a role in professional medical institutions. However, they do exist and have a detrimental effect on the quality of the judgments rendered on any individual case. Moreover, noise is a phenomenon largely independent of the effort to eliminate bias.

Regarding the second problem, there is currently no consensus on diagnostic AI tools and systems for performing genuine abductions. Machines currently do not perform genuine abductions in terms of producing novel ideas about diagnoses. They do not make true discoveries. Their role is to support and error-correct the process of medical inquiry that moves from the deductive to the inductive stages of the diagnostic process. A medical diagnosis involves making a prediction in the form of judgment. Machines do not draw judgments; they compute predictions.

Representing information, language, case studies, and statistical data and their correlations in multiple modalities fosters human imagination and hence serves to trigger abductions. AI diagnostics can contribute to improvements in the skill and accuracy of medical reasoning at both ends of the two spectra of clinical knowledge creation: the human and the machine and the practitioner and the patient. As representations of medical data across various modalities, including kinematic mental simulations, can facilitate the skilful exercise of human judgment, so can improvements in the correctness of human predictions when algorithmic decision-making is being utilized [25, 26, 32,60: 124]. AI tools are thus integral parts of the validation and support of clinical beliefs that translate diagnostics to positive therapeutic choices [29, 39, 61]. Unlike human reasoners, expert machines are not subject to stress, fatigue, mood, or cognitive blind spots and biases hampering professional judgment. They outperform human accuracy and improve diagnostic quality in institutionalized human-AI interactions. However, unlike with human physicians, machine predictions bring forth no elements of genuine novelty, intention, or meaning in their otherwise human-surpassing mechanical approach to diagnostics.

## Summary: the relevance of pragmatism in the philosophy of medical diagnostics

We briefly rephrase the previous observations in terms of our preferred underlying philosophy of clinical reasoning, which is pragmatism. Historically, pragmatism is the only original American philosophy and seems to have gotten to the heart of the matter of reasoning in the following senses. Here, we summarize the relevant views of the big three: William James, John Dewey, and Charles Peirce.

William James (1842–1910) famously posited that the knower is an active participant in the creation of truth and “not simply a mirror floating with no foot-hold anywhere, and passively reflecting an order that he comes upon and finds simply existing. The knower is an actor, and the truth that he helps to create” ([24] 1). The concept of ‘action-first knowledge’, which we find to be a common theme with the original statements of pragmatism, is equally applicable in clinical contexts where physicians actively engage in the diagnostic process, shaping the understanding and treatment of diseases in a threefold interaction with the beneficiaries of the cases presented (the patients) and the contexts of the decisions and choices (the medical institutions, teams, and cultures of organisations).

In the words of John Dewey (1859–1951), humans (patients) are thrown in a problematic situation [17, 18] that in our setting is a situation that is framed and defined by the onset of symptoms. Theoretically, a clinically relevant problematic situation is a deviation from a baseline health situation in both pre- and asymptomatic patients. The resolution of the problematic situation aims at ameliorating the patient’s experience, which only partially overlaps with the idea of healing as the resolution of the underlying causes. Thus, the treatment-oriented perspective also begins *with res media*, with the problematic situation presented to healthcare professionals to be addressed from physicians’ and their institutions’ points of view.

Third, in terms of our interpretation of Charles Peirce’s (1839--1914) theory of the logic of science, *every medical-care operation is a logical operation of thought*. It begins with abduction, which aims at producing a question or recommendation to keep inquiry alive and well, just as the medical reasoning in its full sense is intended to improve the patient’s problematic situation. When performing with adequate skills, making an informed guess by what Peirce termed abductive reasoning, and although abduction is a highly vulnerable and fallible form of reasoning, it works at above-chance levels. Despite medical professionals having to engage in professional guesswork in their daily practice, diagnostic reasoning that begins with abductions is not a random, let alone irrational, process. It is constrained by prior knowledge, beliefs, and values present in any situation in which reasoning is exercised: indeed, logic, according to Peirce [40], “is rooted in the social principle”.

It follows that much of what is referred to in the current literature as “tacit”, “nonanalytical”, “experiential”, “hunch-like” or “gut-feeling” types of intuitive, System 1 cognitive processes are in fact at the bottom addressable as elements of logical, i.e., abductive, reasoning processes. However, as with any highly fallible and nonsecure type of reasoning, this is not a diagnostic space where intuitions roam free [66]. In contrast, delaying intuitions is a savvy thing to do at the initial stages of abductions that begin with the mind’s “play of musement”. It is the free play of ideas focused on *noticing*: turning up our aesthetic sensitivity to those qualities that can be of prime importance to the resolution of the problematic case. Abductions work well only when one already has deep insights into problematic situations and the physician can recognize the nature of the ‘surprise’ presented in each given case, as opposed to running the cases through a general schema of decisions that are the likely ones in terms of being based on observations, judgments, and known probability distributions.

Successful diagnostics is a skill- and inquiry-driven practice aimed at true discovery. It occurs in the context of professional institutions exerting ability and expertise at various levels and capacities. Thus, the institutions likewise are operating in the middle of things, in Dewey's problematic situations in which medical cases are presented and resolved with varying success—the situation that serves to trigger the back-and-forth between doubts and beliefs in the minds of the patients, their healthcare professionals, and in the habits, cultures, and operations of the institutions.

## Conclusions

Theoretically, the deep insights of pragmatist philosophers from the late nineteenth century and early twentieth century were later thematized as theories of situativity, distributed cognition, and related context-sensitive accounts of clinical reasoning that take lessons from contemporary cognitive psychology as well as pragmatism [7, 12, 28]. In the present paper, we have argued that clinical reasoners find their thinking both constrained and facilitated by personal and institutional routines of acting and reasoning. Professionals think less in terms of the ‘social’ (in the sense of the ‘social’ meaning sources of unwanted noise in decision making) aspects of diagnostic reasoning, as they acknowledge that medical knowledge is institutional in its positive valence, as long as those institutions are what Rauch [49] aptly termed ‘reality based’.

In medical institutions, debiasing efforts have been relatively successful. However, much less has been achieved in reducing noise (PubMed currently lists exactly one reference to [25]). Means of noise reduction exist that are resilient and resistant to amplifying noise and errors in institutionalized medical reasoning. Medical models of good reasoning in the clinic resemble the types of slow thinking characteristic of strong institutions that sustain their operations in disinterested and autonomous fashion: they reduce individual noise by delaying institutional ‘intuitions’ and combat group-level noise by averaging over individual differences by statistical correction and averaging methods in their decision hygiene and noise audit protocols and can increasingly achieve this by wisely adopting AI diagnostic support tools.

In the present paper, we addressed the question of improving both the accuracy and skill in clinical reasoning in terms of the three-stage theory of logical reasoning that originates from Peirce, consisting of abduction, deduction, and induction. We have emphasized that in the institutionalized context of medical diagnostics, the power of abduction stems from individual reasoners’ state of mind and mental skills, which are both sensitive and attuned to the reality of the case, with the least number of cognitive distractions and sources for noise that affect prediction and judgment. We noticed that AI tools are promising for reducing noise, leading to a decrease in diagnostic errors. In sum, we underscore the pivotal role of abduction, deduction, and induction in the fabric of medical reasoning within institutional clinical practices, highlighting the indispensable nature of abduction in hypothesis generation, the rigor of deduction in hypothesis testing, and the empirical grounding of induction in validating judgments rendered in clinical decisions.

## Data Availability

No datasets were generated or analysed during the current study.
